# The complete chloroplast genome sequence of *Caryodaphnopsis tonkinensis*

**DOI:** 10.1080/23802359.2019.1698997

**Published:** 2019-12-12

**Authors:** Yuan Zheng, Yana Luo, Yunqing Li, Yi Wang

**Affiliations:** a Laboratory of Forest Plant Cultivation and Utilization, Yunnan Academy of Forestry, Kunming, People’s Republic of China;; b College of Forestry, Southwest Forestry University, Kunming, Yunnan, People’s Republic of China

**Keywords:** *Caryodaphnopsis tonkinensis*, chloroplast, Illumina sequencing, phylogenetic analysis

## Abstract

The first complete chloroplast genome (cpDNA) sequence of *Caryodaphnopsis tonkinensis* was determined from Illumina HiSeq pair-end sequencing data in this study. The cpDNA is 149,016 bp in length, contains a large single copy region (LSC) of 91,915 bp and a small single copy region (SSC) of 17,703 bp, which were separated by a pair of inverted repeats (IR) regions of 19,699 bp. The genome contains 126 genes, including 81 protein-coding genes, eight ribosomal RNA genes, and 36 transfer RNA genes. The overall GC content of the whole genome is 39.0%, and the corresponding values of the LSC, SSC, and IR regions are 37.7%, 34.4%, and 44.4%, respectively. Further phylogenomic analysis showed that *C. tonkinensis* clustered in a clade in Lauraceae family.

*Caryodaphnopsis tonkinensis* is the species of the genus *Caryodaphnopsis* within the family Lauraceae (Zeng et al. 2014). It distributes in southern Yunnan of China, Vietnam, Malaysia, and the Philippines (Li et al. 2016). *Caryodaphnopsis tonkinensis* usually grows in the valley and forest edge and it is very important for classification, systemic evolution and karst ecosystems (Richter 1980; Tuyet 2001). However, there has been no genomic studies on *C. tonkinensis*.

Herein, we reported and characterized the complete *C. tonkinensis* plastid genome (MN698962). One *C. tonkinensis* individual (specimen number: 5309270372) was collected from Cangyuan, Yunnan Province of China (23°15′14″N, 99°02′50″E). The specimen is stored at Yunnan Academy of Forestry Herbarium, Kunming, China, and the accession number is YAFH0012761. DNA was extracted from its fresh leaves using DNA Plantzol Reagent (Invitrogen, Carlsbad, CA, USA).

Paired-end reads were sequenced by using Illumina HiSeq system (Illumina, San Diego, CA). In total, about 21.6 million high-quality clean reads were generated with adaptors trimmed. Aligning, assembly, and annotation were conducted by CLC de novo assembler (CLC Bio, Aarhus, Denmark), BLAST, GeSeq (Tillich et al. 2017), and GENEIOUS v 11.0.5 (Biomatters Ltd, Auckland, New Zealand). To confirm the phylogenetic position of *C. tonkinensis*, other fourteen species of *Lauraceae* family from NCBI were aligned using MAFFT v.7 (Katoh and Standley 2013). The Auto algorithm in the MAFFT alignment software was used to align the fifteen complete genome sequences and the G-INS-i algorithm was used to align the partial complex sequences. The maximum-likelihood (ML) bootstrap analysis was conducted using RAxML (Stamatakis 2006); bootstrap probability values were calculated from 1000 replicates. *Chimonanthus nitens* (MH377058) and *Chimonanthus praecox* (MH377057) were served as the out-group.

The complete *C. tonkinensis* plastid genome is a circular DNA molecule with the length of 149,016 bp, contains a large single copy region (LSC) of 91,915 bp and a small single copy region (SSC) of 17,703 bp, which were separated by a pair of inverted repeats (IR) regions of 19,699 bp. The overall GC content of the whole genome is 39.0%, and the corresponding values of the LSC, SSC, and IR regions are 37.7%, 34.4%, and 44.4%, respectively. The plastid genome contained 126 genes, including 81 protein-coding genes, eight ribosomal RNA genes, and 36 transfer RNA genes. Phylogenetic analysis showed that *C. tonkinensis* clustered in a unique clade in *Lauraceae* family (Figure 1). The determination of the complete plastid genome sequences provided new molecular data to illuminate the *Lauraceae* family evolution.

**Figure 1.**
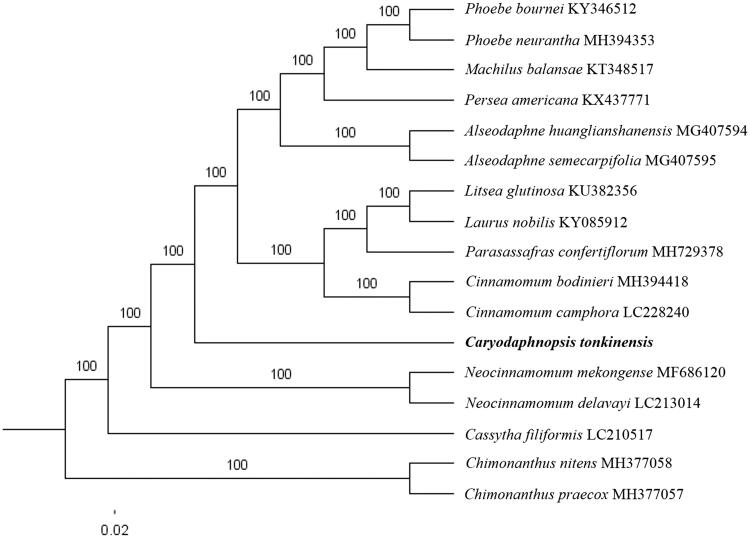
The maximum-likelihood tree based on the 15 chloroplast genomes of *Lauraceae* family. The bootstrap value based on 1000 replicates is shown on each node.
